# Prevalence and Risk Factors of Acute Ischemic Stroke in Patients with Antiphospholipid Syndrome: A Retrospective Monocenter Analysis

**DOI:** 10.3390/jcdd12050183

**Published:** 2025-05-14

**Authors:** Paschalis Evangelidis, Nikolaos Kotsiou, Panagiotis Kalmoukos, Zacharo Ntova, Theodosia Papadopoulou, Sofia Chissan, Anastasia Sarvani, Styliani Kokoris, Elisavet Grouzi, Michael Doumas, Sofia Vakalopoulou, Eleni Gavriilaki

**Affiliations:** 1 Second Propaedeutic Department of Internal Medicine, Aristotle University of Thessaloniki, Hippocration Hospital, 54642 Thessaloniki, Greece; pascevan@auth.gr (P.E.); kotsiounikolaos@gmail.com (N.K.); kalmoukosp@yahoo.gr (P.K.); ntovairo@gmail.com (Z.N.); sissipapth@yahoo.gr (T.P.); sofiachissan@yahoo.com (S.C.); natasasar91@gmail.com (A.S.); michalisdoumas@yahoo.co.uk (M.D.); svakalopoulou@yahoo.com (S.V.); 2 Laboratory of Hematology and Blood Bank Unit, “Attikon” University General Hospital, Medical School, National and Kapodistrian University of Athens, 12462 Athens, Greece; stylianikok@gmail.com; 3 Department of Transfusion Service and Clinical Hemostasis, “Saint Savvas” Oncology Hospital, 11522 Athens, Greece; grouzielisavet@gmail.com

**Keywords:** acute ischemic stroke, aGAPSS, antiphospholipid syndrome, hypertension, thrombosis

## Abstract

(1) Background: Antiphospholipid syndrome (APS) is associated with thrombotic events and the laboratory identification of antiphospholipid antibodies (aPL), in which lupus anticoagulant (LA), anticardiolipin (aCL), and anti-β2 glycoprotein I antibodies are included. The aim of the current retrospective study is to examine clinical characteristics and risk factors of ischemic stroke as a clinical manifestation of APS. (2) Methods: Adult patients diagnosed with APS between 1 January 2009 and 1 June 2024 were retrospectively enrolled in this study. Sydney-revised Sapporo criteria were used for the diagnosis of APS, while ischemic stroke was diagnosed based on the acute onset of focal neurologic deficits and confirmed with radiological findings. (3) Results: We studied 115 patients with APS. Specifically, 28 (24.35%) patients, with a mean age (standard deviation) of 54 (±12.5), had ischemic stroke as a clinical manifestation of APS. In univariate analysis, stroke development was associated with the following factors: age (*p* < 0.001), livedo reticularis (*p* = 0.046), avascular necrosis (AVN) (*p* = 0.046), hypertension (*p* < 0.001), dyslipidemia (*p* = 0.013), aCL IgG (U/L) antibodies title (*p* = 0.035), and adjusted global APS score (aGAPSS) (*p* = 0.047), while in multivariate analysis, it was associated with age (*p* = 0.006), hypertension (*p* < 0.001), AVN (*p* = 0.006), livedo reticularis (*p* = 0.035), aCL IgG title (*p* = 0.004), and aGAPSS (*p* = 0.002). (4) Conclusions: Stroke is a common initial manifestation of APS, with cardiovascular risk factors, particularly hypertension, being highly prevalent.

## 1. Introduction

Antiphospholipid syndrome (APS) is a systemic autoimmune disorder that is characterized by the persistent presence of antiphospholipid antibodies (aPL) combined with thrombotic events, obstetric complications, and other non-thrombotic manifestations [1]. APS can be categorized as primary or secondary, with secondary APS occurring in the context of another autoimmune disorder, most commonly systemic lupus erythematosus (SLE) [2]. The pathogenesis of this clinical entity includes multiple interactions between aPL, endothelium, coagulation cascade, and complement system [3]. Thrombosis can be either arterial, venous, or microvascular, leading to the development of thrombotic microangiopathy [4]. The most common sites of thrombosis include the deep veins of the lower extremities and cerebral arteries, while cases of thrombosis in unusual sites can also be observed. Other manifestations of APS are considered as thrombocytopenia, pulmonary hemorrhage, aPL nephropathy, and heart valve disease [5,6].

The up-to-date diagnosis of APS is based on the revised Sydney–Sapporo Criteria [7]. Recently, the 2023 ACR/EULAR antiphospholipid syndrome criteria have been suggested for the classification of APS patients [8]. Vitamin K antagonists remain the cornerstone of management for most patients with thrombotic APS [9]. Other agents used in the treatment of APS include low-dose aspirin, hydroxychloroquine (HCQ), and prednisone [9]. Catastrophic APS is characterized by micro- and macro-thrombosis in multiple sites, organ failure, and high mortality and morbidity. The differential diagnosis of this syndrome from other clinical entities, such as thrombotic microangiopathies, is challenging [10]. Moreover, novel agents such as complement inhibitors have been reported as beneficial for some patients [11].

The most common clinical manifestation of APS-associated arterial thrombosis is considered acute ischemic stroke [12]. At the same time, APS is an important etiology of stroke in individuals below the age of 45 years [13]. In addition to cerebrovascular disease, including acute ischemic stroke, transient ischemic attacks, and cerebral venous thrombosis, neurological manifestations of this condition include cognitive deficits, migraines, transverse myelitis, chorea, psychosis, and Sneddon syndrome [14]. Based on the common involvement of the central nervous system in APS patients and the burden of disease that stroke survivors experience, the aim of this study is to investigate the prevalence and risk factors associated with acute ischemic stroke in a real-world cohort of APS patients.

## 2. Materials and Methods

### 2.1. Study Design and Patient Population

In this study, adult patients (both inpatients and outpatients) with definite diagnoses of APS who visited the thrombosis–hemostasis referral clinic of the 2nd Propedeutic Department of Internal Medicine between 1st January 2009 and 1st June 2024 were enrolled. APS diagnosis was based on the revised Sydney–Sapporo Criteria [7]. Specifically, a diagnosis of APS was made when at least one of the clinical criteria and one of the laboratory criteria were present. Clinical criteria included vascular thrombosis and pregnancy morbidity, while the laboratory criteria were based on the presence of lupus anticoagulant (LA) in plasma, anticardiolipin antibody (aCL) of IgG, and/or the IgM isotype in serum or plasma, in medium or high titer, and the anti-b2 glycoprotein-I antibody (aβ2GPI) of IgG and/or IgM isotype in serum or plasma on two or more occasions at least 12 weeks apart. The study was conducted according to the Declaration of Helsinki and was approved by the Institutional Review Board of Hippocration General Hospital, with informed consent collected from all participants.

The following data were retrospectively collected from the patient’s medical records: gender, age at APS diagnosis, previous history of thrombosis, thrombocytopenia (platelet counts < 100 × 10^9^/L), neuropsychiatric manifestations, other APS manifestations (such as history of avascular necrosis [AVN] or livedo reticularis), cardiovascular disease risk factors (arterial hypertension, dyslipidemia, diabetes mellitus), underlying autoimmune disease, homocysteine levels, thrombophilia genetic testing (factor V [FV] Leiden, factor II [FII] (G20210A) mutation, methylenetetrahydrofolate reductase [MTHFR] mutation), LA activity and positivity, aCL title and positivity, aβ2GPI title and positivity, and antinuclear antibody (ANA) positivity.

Acute ischemic stroke was diagnosed by experienced neurologists based on the patient’s history, clinical condition, and neuroimaging (magnetic resonance imaging [MRI] or computed tomography [CT]) findings. Data regarding follow-up visits were obtained from the patient’s records, while venous thromboembolism was diagnosed based on clinical manifestations and radiological imaging.

### 2.2. Detection of aPLs and aGAPSS Calculation

aCL and aβ2GPI were detected with the use of ELISA kits (EUROIMMUN, Luebeck, Germany). The aCL and aβ2GPI threshold for positivity was ≥40 units based on standardized ELISA results [8]. LA was measured according to the recommendations of ISTH, using diluted Russel’s viper venom time [15]. All patients diagnosed with APS had positive aPLs during at least 2 time points within an interval of a minimum of 12 weeks. Additionally, the adjusted global antiphospholipid syndrome score (aGAPSS) was retrospectively calculated based on the presence of dyslipidemia (3 points), arterial hypertension (1 point), aCL IgG/IgM antibodies (5 points), aβ2GPI IgG/IgM (4 points), and LA (4 Points) [16].

### 2.3. Statistical Analysis

The sample size calculation was based on previously published studies on stroke in APS patients [17,18]. Statistical analysis was performed with SPSS 28.0 (IBM SPSS Statistics for Windows, Version 28.0. Armonk, NY, USA: IBM Corp). Frequencies were used for the presentation of categorical variables. Shapiro–Wilk and Kolmogorov–Smirnov tests (based on the sample size for each time) were employed for the determination of the sample’s normality distribution. Normally distributed variables were presented as means with standard deviation (±SD), while those with non-normal distribution were presented as medians with an interquartile ratio (IQR: Q1–Q3). For categorical variables, chi-squared or Fisher’s exact tests, when required, were used to analyze the differences between the groups. For continuous variables, Student’s *t*-test (for normally distributed variables) and the Mann–Whitney U test (for non-normally distributed) were employed to identify differences. Univariate logistic regression analysis was used for the identification of potential factors associated with stroke development. Variables with a significance level of *p* < 0.05 were included in multivariate logistic regression models, along with gender. Odds ratios (ORs) and corresponding 95% confidence intervals (CIs) were estimated. The Hosmer–Lemeshow test was used to examine the multivariate logistic regression model’s goodness of fit and calibration. The fit was considered poor if the significance value was less than 0.05. The statistical significance level was set to *p* < 0.05.

## 3. Results

### 3.1. Clinical and Laboratory Characteristics of APS Patients with Stroke

**Figure 1:**
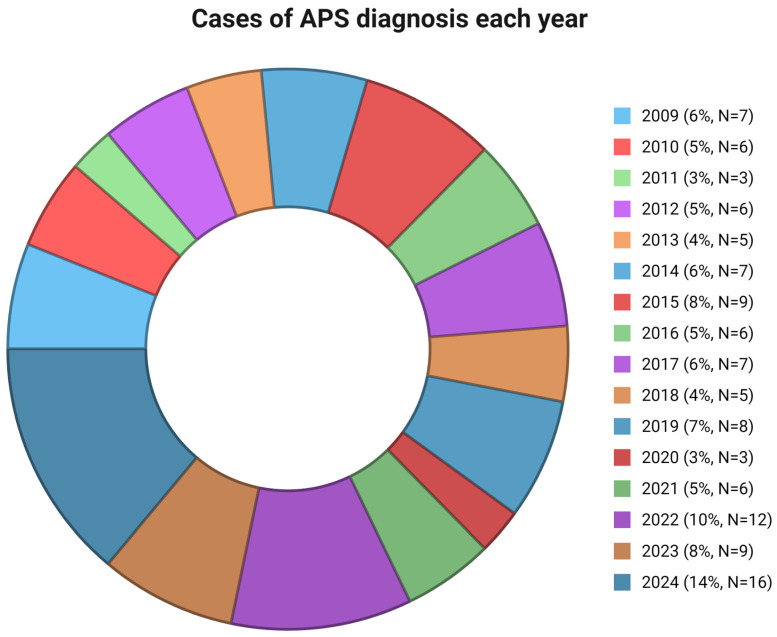
Cases of APS diagnosis each year. APS: antiphospholipid syndrome.

Among 115 patients with APS, 28 (24.35%) presented with acute ischemic stroke. In Figure 1, the cases of APS diagnosed each year from 2009 to 2024 are presented. The mean age at diagnosis was 54 (±12.5) years, significantly higher compared to patients without acute ischemic stroke (41.5 ± 14.3) (*p* < 0.001), while 15 (53.6%) APS patients were also female. Four (14.28%) patients had a previous history of thrombosis, three (10.7%) of AVN of the femoral head, and three (10.7%) of livedo reticularis. Moreover, AVN (*p* = 0.043) and livedo reticularis (*p* = 0.043) were more frequent in the stroke group in comparison to other patients. Thrombocytopenia at diagnosis was observed in one (3.6%) patient with stroke and neuropsychiatric manifestations were shown in two patients (7.2%, migraine and cognitive dysfunction in both).

Regarding cardiovascular disease risk factors, 15 (53.6%) stroke patients had a previous medical history of arterial hypertension, seven (25%) of dyslipidemia, and one (3.6%) of diabetes mellitus. Arterial hypertension (*p* < 0.001) and dyslipidemia (*p* = 0.008) were more common in the stroke group compared to patients without stroke. Furthermore, three individuals (10.7%) were diagnosed with secondary APS due to underlying SLE. The three patients with SLE in the stroke group were treated with HCQ, while among the eight other SLE patients without stroke, five received HCQ and three HCQ in combination with other immunosuppressant agents.

Three (10.7%) patients were heterozygous for the FV Leiden mutation, while one (3.6%) had the FII (G20210A) mutation. Furthermore, all patients with ischemic stroke were positive for LA, 12 (42.9%) for aCLs, and 13 (46.4%) for aβ2GPI, respectively. Triple positivity was observed in eight (28.6%) individuals, while ANA positivity was detected in eleven (39.3%). The median aGAPSS score was nine (4–13.75), which was higher in stroke patients compared to others (*p* = 0.048). The clinical and laboratory characteristics of APS patients with ischemic stroke in comparison to those without stroke are summarized in Table 1. All APS patients with stroke were treated with vitamin K antagonists. Follow-up data were available for seventeen stroke patients, and the recurrence of thrombosis (venous thromboembolism in all cases) was observed in three (17.65%) of them.

### 3.2. Univariate Analysis

In univariate analysis, ischemic stroke in patients with APS was associated with the following factors: age at diagnosis (OR: 0.938, 95% CI: −0.520, −0.191, *p* < 0.001), livedo reticularis (OR: 10.44, 95% CI: 0.044, 0.391, *p* = 0.046), AVN of the femoral head (OR: 10.44, 95% CI: 0.044, 0.391, *p* = 0.046), arterial hypertension (OR: 15.769, 95% CI: 0.373, 0.641, *p* < 0.001), dyslipidemia (OR: 4.556, 95% CI: 0.067, 0.410, *p* = 0.013), title of aCL IgG (OR: 0.975, 95% CI: −0.031, −0.033, *p* = 0.035), and aGAPSS (OR: 0.916, 95% CI: −0.360, −0.003, *p* = 0.047). The findings of the univariate analysis are presented in Table 2.

### 3.3. Multivariate Analysis

In the multivariate analysis, ischemic stroke was associated with age at diagnosis (OR: 0.925, 95% CI: 0.876, 0.977, *p* = 0.006), livedo reticularis (OR: 0.017, 95% CI: 0.000, 0.755, *p* = 0.035), AVN (OR: 228.573, 95% CI: 4.684, 11,153.959, *p* = 0.006), arterial hypertension (OR: 608.982, 95% CI: 20.519, 18,073.990, *p* < 0.001), aCL IgG title (OR: 0.916, 95% CI: 0.863, 0.973, *p* = 0.004) and aGAPSS (OR: 1.899, 95% CI: 1.263, 2.854, *p* = 0.002). In Table 3, the findings of the multivariate regression analysis are summarized.

## 4. Discussion

In this retrospective real-world study, 115 patients with APS were examined. Acute ischemic stroke (28 patients, 24.35%) was found as a common manifestation of APS. Moreover, age, livedo reticularis, AVN, aGAPSS, and cardiovascular disease risk factors, including arterial hypertension and dyslipidemia, were associated with stroke development in these patients. Furthermore, cases of APS diagnosis each year during the study period were presented. It is well known that the COVID-19 pandemic had a significant impact on risk factors associated with acute ischemic stroke [19,20]. However, in our real-world study, the number of patients referred to our center with APS was relatively small. This can be attributed to barriers in healthcare access during the pandemic that limited patients’ access to specialized centers such as ours [21].

The findings of our study were in line with previously published data in this field. García-Grimshaw et al. reported, in their retrospective study of 120 cases of APS with cerebrovascular events, that acute ischemic stroke was the most common type of these events [22]. In the study by Liu et al., 342 APS patients were enrolled, and neurological manifestations were recorded in 119 (34.8%) individuals [18]. In their analysis, older age (*p* = 0.047), livedo reticularis (*p* = 0.038), and dyslipidemia (*p* = 0.030) were correlated with central nervous system manifestations. Similarly, in our study, age at diagnosis, livedo reticularis, AVN, arterial hypertension, and dyslipidemia were associated with ischemic stroke [18]. Additionally, in the cohort of Fan et al., stroke was reported in 25.8% (93/361) of the participants, while in the multivariate regression analysis, arterial hypertension, diabetes mellitus, livedo reticularis, and other neurological manifestations were significantly related to stroke development [17]. In the cross-sectional study by Djokovic et al., it was shown that male gender and the presence of aβ2GPIs IgG were related significantly to stroke [23]. We showed that stroke development in APS patients was associated with the title of aCL IgG. Urbanski et al. compared the characteristics of APS patients with isolated IgM positivity (aCL or aβ2GPIs) with the non-isolated IgM populations, and they reported that stroke was more frequent in APS patients with isolated APS after adjustment for cardiovascular risk factors (odds ratio, 3.8; 95% CI, 1.3–11.5) [24].

aGAPSS, which can be easily calculated by commonly obtained clinical and laboratory factors, has been developed and validated for the prediction of thrombosis recurrence in APS patients [16]. Additionally, aGAPSS has been associated with coronary artery disease, stroke, and peripheral artery disease in this clinical entity [25]. In the study by Radin et al., the mean aGAPSS was significantly higher in APS patients with stroke compared to those without (*p* < 0.05) [26]. In our patient population, this score was correlated with stroke development in both univariate and multivariate analyses. Song et al. developed aGAPSS–ischemic stroke (aGAPSS-IS), which predicted the development of stroke in APS patients with higher accuracy compared to aGAPSS in both training and validating cohorts. This score is calculated based on the following factors: age (years), presence of diabetes mellitus, platelet count (×10^9^/L), hyperuricemia, and aGAPSS > 10. Future work should focus on the predictive roles of these models in real-world patients.

Gašperšič et al. studied 89 patients with cerebrovascular events, and 22% of them fulfilled the criteria for APS diagnosis [27]. It was found that the persistent positivity of aPLs was an independent risk factor for stroke development (OR: 4.62), while a statistically significant difference in the incidence of thrombosis recurrence was not identified between APS and non-APS patients [27]. Saidi et al. compared aPL levels between patients with stroke and healthy controls [28]. Interestingly, positivity for LA resulted in an increased risk for stroke, while elevated levels of aCLs were identified in patients with lacunar, atherosclerotic, and cardioembolic stroke subtypes [28]. Moreover, Wang and colleagues evaluated aPLs in stroke patients and assessed post-stroke depression development 3 months after stroke onset by using the 24-item Hamilton Depression Rating Scale [29]. It was shown that increased aPL levels at baseline (acute phase of ischemic stroke) were associated with a higher risk of 3-month post-stroke depression [29]. APS has been shown as an independent risk factor for a hemorrhagic transformation of ischemic stroke [30]. Future studies should evaluate the possible associations between aPL type and title ischemic infract and radiological neuroimaging findings.

Ricarte et al., in their observational study, evaluated cerebral hemodynamic factors with transcranial Doppler in 46 patients with APS (22 with primary and 24 with secondary APS) in comparison to healthy controls [31]. They reported that right-to-left circulation shunts were more common in the APS group compared to controls (63.6% versus 38.1%, *p* = 0.05), underlying the importance of cardiac investigation in this patient population [31]. More data regarding hemodynamic alterations and brain microcirculation impairment in APS patients are essential. Interestingly, complement activation is implicated in the pathogenesis of thrombosis in APS, while in catastrophic APS, genetic predisposition to complement dysregulation has been recognized [32,33,34,35]. Markers of complement activation, such as soluble C5b-9 levels and the modified Ham test, as well as their associations with acute ischemic stroke development and severity in APS, should be further evaluated [36,37].

In our real-world cohort, all APS patients with ischemic stroke were treated with vitamin K antagonists. Current guidelines suggest that direct oral anticoagulants (DOACs) should not be used for the prevention of thrombosis in APS patients [9]. Sikorska et al., in their real-world study, studied 152 patients with APS, of whom 66 patients were treated with a DOAC (apixaban) and 86 with warfarin [38]. It was found that APS patients receiving DOAC exhibited a similar risk of thrombosis recurrency in comparison to those on warfarin [38]. In the clinical trial of Ordi-Ros et al., rivaroxaban was compared to dose-adjusted vitamin K antagonists in 190 adult patients with thrombotic APS. In a 3-year follow-up period, recurrent thrombosis was recorded in 11.6% of patients in the rivaroxaban group and six in the vitamin K antagonist arm without a statistically significant difference [39]. In a systematic review and meta-analysis of three clinical trials comparing DOACs with vitamin K antagonists as secondary APS prophylaxis, it was shown that DOACs might increase the risk of stroke in these patients [40]. Nevertheless, more data regarding the specific groups of APS patients who might experience benefits from DOAC use are essential, based both on clinical trials comparing DOACs with vitamin K antagonists but also on real-world data [41].

In a survey conducted by Cohen et al. investigating the attitudes of 107 physicians regarding the diagnosis and treatment of APS, variations were identified in aPL testing practices for brain ischemic injury beyond ischemic stroke or transient ischemic attack in cases where an alternative cause for stroke was present and in the age cutoff for aPL testing in stroke patients [38]. Our study mainly focused on ischemic stroke as a clinical manifestation of APS. However, beyond the thrombotic manifestations of this clinical entity, several other neuropsychiatric manifestations have been described in these patients, including migraines, cognitive dysfunction, seizures, transverse myelitis, multiple sclerosis-like symptoms, and psychiatric symptoms [39]. Notably, cognitive impairment in these patients has been described as a result of complex interactions between the coagulation cascade, immune dysregulation, and endothelial dysfunction [40]. Rosa et al. highlighted that diminished levels of brain-derived neurotrophic factor were correlated with cognitive dysfunction in patients with primary APS (*p* = 0.032) [42]. Other rare cerebrovascular manifestations of APS include the dissection of the cerebral artery, mainly in young adults [43].

Some limitations should be recognized in our research design. Firstly, it was a retrospective study, while the follow-up data were available only for some APS patients. A prospective design would have elucidated the potential biases of a retrospective study. Moreover, it was a monocenter study from a thrombosis and hemostasis referral center in Northern Greece. The study population was relatively small, and control and stroke groups were not age- and gender-matched, thus influencing statistical analysis findings. Multicenter collaboration and studies with a larger number of participants are essential in the field. Another limitation of our study includes the lack of significant cardiovascular disease and venous thromboembolism characteristics of the patients, given its retrospective design, which are incorporated in the new APS criteria [8]. Finally, our study included patients with secondary APS (SLE), given that the underlying autoimmune disease itself might contribute independently to ischemic stroke development.

## 5. Conclusions

In this retrospective monocenter study, acute ischemic stroke was identified as a common clinical manifestation of APS. Factors including age, livedo reticularis, AVN, arterial hypertension, aGAPSS, and dyslipidemia were associated with stroke development. Multicenter collaborative studies can be helpful towards a better understanding of APS-related stroke epidemiology and risk factors. Moreover, the role of endothelial dysfunction, microvascular inflammation, and complement activation and association of these factors with not only thrombotic risk in APS but also with other complications, such as cognitive impairment, should be further evaluated.

## Figures and Tables

**Table 1:** Clinical and laboratory characteristics of APS patients with ischemic stroke in comparison with those without.

	APS Patients with Stroke (N = 28)	APS Patients Without Stroke (N = 87)	*p*
Gender, n (%) Male Female	13 (46.4) 15 (53.6)	29 (33.3) 58 (66.7)	0.211
Mean age at diagnosis (±SD)	54 (12.5)	41.5 (14.3)	<0.001
Median age (IQR)	54 (45–64)	40 (30–53)	NS
Previous history of thrombosis, n (%)	4 (14.28)	15 (17.3)	1.000
Avascular necrosis, n (%)	3 (10.7)	1 (1.2)	0.043
Livedo reticularis, n (%)	3 (10.7)	1 (1.2)	0.043
Thrombocytopenia, n (%)	1 (3.6)	8 (9.1)	0.685
Neuropsychiatric manifestations, n (%)	2 (7.2)	1 (1.2)	0.144
Arterial hypertension, n (%)	15 (53.6)	6 (6.9)	<0.001
Dyslipidemia, n (%)	7 (25)	6 (6.9)	0.008
Diabetes mellitus, n (%)	1 (3.6)	3 (3.5)	1.000
Secondary APS, n (%)	3 (10.7)	8 (9.2)	0.725
Hyperhomocysteinemia, n (%)	5 (17.9)	9 (10.4)	0.280
FV Leiden mutation, n (%)	3 (10.7)	7 (8)	0.6971
FII (G20210A) mutation, n (%)	1 (3.6)	4 (4.6)	1.000
MTHFR (C677T) mutation, n (%)	5 (17.9)	14 (16.1)	0.827
LA positivity, n (%)	28 (100)	81 (93.1)	0.124
aCL positivity, n (%)	12 (42.9)	26 (29.9)	0.191
aβ2GPI positivity, n (%)	13 (46.4)	27 (31)	0.127
Single aPL positivity, n (%)	15 (53.6)	61 (70.1)	0.127
Double aPL positivity, n (%)	5 (17.9)	13 (15)	
Triple aPL positivity, n (%)	8 (28.6)	13 (14.9)	0.099
ANA positivity, n (%)	11 (39.3)	35 (40.2)	0.507
Median aGAPSS (IQR)	9 (4–13.75)	8 (4–9)	0.048
Mean C3 levels (mg/dL) (+SD)	115.1 (±47.8)	115.9 (±30.4)	0.953
Median C4 levels (mg/dL) (IQR)	23.3 (16.3–27.1)	21.8 (16.4–28.5)	1.000

aCL: anticardiolipin antibodies; aGAPSS: adjusted global antiphospholipid syndrome score; ANA: antinuclear antibodies; aPL: antiphospholipid antibodies; APS: antiphospholipid syndrome; aβ2GPI: anti-β2 glycoprotein I antibodies; C3: complement factor 3; C4: complement factor 4; dl: deciliter; FII: factor II; FV: Factor V; IQR: interquartile ratio; LA: lupus anticoagulant; MTHFR: methylenetetrahydrofolate reductase; mg: milligram; NS: not stated; SD: standard deviation.

**Table 2:** Univariate analysis of factors associated with ischemic stroke in APS patients.

	OR	95% CI	*p*
Age at diagnosis	0.938	−0.520, −0.191	<0.001
Livedo reticularis	10.44	0.044, 0.391	0.046
Avascular necrosis	10.44	0.044, 0.391	0.046
Arterial hypertension	15.769	0.373, 0.641	<0.001
Dyslipidemia	4.556	0.067, 0.410	0.013
aCL IgG title	0.975	−0.031, −0.033	0.035
aGAPSS	0.916	−0.360, −0.003	0.047

aCL: anticardiolipin antibodies; aGAPSS: adjusted global antiphospholipid syndrome score; APS: antiphospholipid syndrome; CI: confidence interval; OR: odds ratio.

**Table 3:** Multivariate analysis of factors associated with ischemic stroke in APS patients.

Hosmer–Lemeshow Test: *p* = 0.308, x^2^ = 9.416			
	OR	95% CI	*p*
Gender	1.171	0.284, 4.836	0.827
Age at diagnosis	0.925	0.876, 0.977	0.006
Livedo reticularis	0.017	0.000, 0.755	0.035
Avascular necrosis	228.573	4.684, 11,153.959	0.006
Arterial hypertension	608.982	20.519, 18,073.990	<0.001
Dyslipidemia	1.062	0.182, 6.203	0.947
aCL IgG title	0.916	0.863, 0.973	0.004
aGAPSS	1.899	1.263, 2.854	0.002

aCL: anticardiolipin antibodies; aGAPSS: adjusted global antiphospholipid syndrome score; APS: antiphospholipid syndrome; CI: confidence interval; OR: odds ratio.

## Data Availability

The authors declare that the data supporting the findings of this study are available within the paper.

## Abbreviations

The following abbreviations are used in this manuscript:

aGAPSS	Adjusted global antiphospholipid syndrome
aGAPSS-IS	aGAPSS–ischemic stroke
aβ2GPI	Anti-b2 glycoprotein-I antibody
aCL	Anticardiolipin antibody
ANA	Antinuclear antibodies
aPL	Antiphospholipid antibodies
APS	Antiphospholipid syndrome
AVN	Avascular necrosis
CT	Computed tomography
CI	Confidence interval
DOAC	Direct oral anticoagulant
FII	Factor II
FV	Factor V
HCQ	Hydroxychloroquine
IQR	Interquartile ratio
LA	Lupus anticoagulant
MRI	Magnetic resonance imaging
MTHFR	Methylenetetrahydrofolate reductase
OR	Odds ratio
SD	Standard deviation
